# Delivery of Timely Adjuvant Radiation Among Veterans With Head and Neck Cancer

**DOI:** 10.1002/oto2.70037

**Published:** 2025-02-06

**Authors:** Jasmine Gulati, Anuja Shah, Veranca Shah, Thomas Haupt, Amanda Walsh, Jessica H. Maxwell

**Affiliations:** ^1^ Department of Otolaryngology MedStar Georgetown University Hospital Washington District of Columbia USA; ^2^ Department of Otolaryngology Howard University College of Medicine Washington District of Columbia USA; ^3^ Department of Otolaryngology Pittsburgh Veterans Affairs Medical Center Pittsburgh Pennsylvania USA; ^4^ Department of Otolaryngology–Head and Neck Surgery University of Pittsburgh Medical Center Pittsburgh Pennsylvania USA

**Keywords:** head and neck squamous cell carcinoma, postoperative radiation therapy, Veteran's Health Care

## Abstract

**Objective:**

The objective of this study was to determine the rate at which veterans with head and neck squamous cell carcinoma (HNSCC) received care adhering to National Comprehensive Care Network (NCCN) guidelines for postoperative radiation and to identify factors associated with non‐adherence. The guidelines recommend initiation of postoperative radiation therapy (PORT) within 6 weeks of surgery.

**Study Design:**

This was a retrospective cohort analysis of a pre‐existing database.

**Setting:**

This study, performed at the DC Veterans Affairs Medical Center, identified patients with HNSCC who underwent surgery with curative intent followed by adjuvant radiation ± chemotherapy between 1991 and 2021.

**Methods:**

Variables assessed included patient demographics, cancer stage and site, treatment type, dates of treatment initiation and completion, and adherence to the NCCN guidelines for PORT initiation. Fisher exact test was used to identify factors associated with delays >6 weeks.

**Results:**

Among the 132 veterans identified, 72 (54.5%) underwent surgery followed by adjuvant PORT. Only 18 veterans (25%) started radiation within 6 weeks of surgery. Patients who underwent a neck dissection at the time of the index surgery (*P* = .028), dental extraction (*P* = .032), or gastrostomy tube placement (*P* = .041) were more likely to experience delays.

**Conclusion:**

Only 25% of veterans initiated PORT within 6 weeks. Identifying causes of delays provides an important step in addressing discrepancies between guideline‐directed care and actual care delivered. Development of efficient care pathways to increase guideline‐congruent initiation of PORT should be considered.

Head and neck squamous cell carcinoma (HNSCC) is a heterogenous group of malignancies that originates in the mucosal epithelial lining of the upper aerodigestive tract, encompassing regions such as the oral cavity, oropharynx, larynx, hypopharynx and paranasal sinuses. HNSCC is the seventh most common cancer globally, and in the United States, about 65,000 patients are diagnosed with HSNCC annually, with a 3:1 male to female gender ratio.^1^ On average, the 5‐year survival rate for all stages of HNSCC combined is around 50% to 60% with a reported 14,600 deaths per year. It is a notoriously aggressive malignancy that is associated with frequent recurrence and, if untreated, debilitating functional morbidities including impairment of speech, swallowing and breathing.

Evidence‐based treatment of locally advanced HNSCC often consists of a combination of surgery, radiation therapy, and/or chemotherapy.^2^ In patients who require postoperative radiation therapy (PORT), the National Comprehensive Care Network (NCCN) guidelines recommend initiating this treatment with or without concurrent chemotherapy within 6 weeks of the initial surgical resection. An increased surgery‐to‐radiation duration is correlated with increased morbidity and mortality, highlighting the importance of timely care.^3^ Delays in initiation of PORT result in proliferation of tumor cells and may allow for metastasis beyond the initial tumor site, resulting in decreased local control by 0.09% for each additional day between surgery and radiation.^4^, ^5^ Given the association between timely PORT and improved overall and recurrence‐free survival, this surgery‐to‐radiation interval has been suggested as a quality indicator in the treatment for patients with HNSCC.^3^

In a study by Graboyes et al evaluating patients with HNSCC who underwent curative‐intent surgery followed by PORT, the rate of adherence to the NCCN guidelines for PORT was just 44.3%.^6^ In this cohort, delays in initiation of PORT disproportionately impacted patients with lower socioeconomic status, racial and ethnic minorities, and uninsured individuals.^7^ However, few studies have evaluated adherence to the NCCN guidelines in a veteran population. Nationally, the Veterans Administration (VA) has faced criticism regarding delivery of timely care, and prior studies have shown that veterans often face significantly longer times to treatment initiation after cancer diagnosis.^8^

In light of this, the primary aim of this study was to assess the level of compliance with NCCN guidelines for PORT in a population of veterans with HSNCC and to identify factors that may impact the delivery of timely care in this population. Understanding these factors can potentially lead to more efficient patient care pathways to increase timely and guideline‐congruent initiation of PORT in United States veterans.

## Methods

This was a retrospective cohort analysis that received Institutional Review Board approval from the DC Veterans Affairs Medical Center Institutional Review Board (Study Number: 01783). We identified patients with HNSCC who underwent treatment with surgery followed by adjuvant radiation ± chemotherapy at the Washington, DC Veterans Affairs Medical Center (VAMC) between 1991 and 2021 using the VA Tumor Registry. For this cohort, the following variables were assessed: clinical and demographic data, treatment types, and dates of treatment initiation and completion. Veterans were grouped into those who adhered to the current NCCN guidelines to begin adjuvant PORT within 6 weeks and those who did not, and factors associated with delays greater than 6 weeks were identified.

Continuous data was presented as means with standard deviations, while categorical data was presented as percentages. Continuous variables were assessed for normality versus skewness and for the presence of outliers. To mitigate skewness, a log transformation was applied, and outliers were recoded to eliminate gaps in the frequency histogram. Continuous variables were analyzed using either the Kruskal‐Wallis test for nonparametric results or 2‐sided *t* tests for parametric data. Categorical variables were analyzed using Fisher's exact test. A 2‐sided *P* value less than .05 was considered statistically significant. Data were analyzed using Stata (StataCorp LLC 2021, Stata Statistical Software: Release 17).

## Results

A total of 320 patients were identified with HNSCC between 1991 and 2021. Of these 320, 132 veterans received curative intent surgery. Of these 132, 72 (54.5%) veterans underwent adjuvant radiation or chemoradiation therapy. Patients in this cohort had a median age of 63 years (±9.7 years) and 97% were male (Table 1).

**Table 1:** Patient Demographics and Comorbidities

	No. of patients, %
Patient variable	Total patients (n = 72)
Sex	
Male	70 (97.2)
Female	2 (2.8)
Race	
White	34 (47.2)
Black	38 (52.8)
Other	0 (0)
Age, y	
<40	0 (0)
40‐49	4 (5.6)
50‐59	29 (40.3)
60‐69	25 (34.7)
70‐79	13 (18.1)
>80	1 (1.4)
BMI	
<18.5	11 (15.3)
18.5‐24.9	29 (40.3)
25‐29.9	23 (31.9)
30‐34.9	7 (9.7)
35‐39.9	2 (2.8)
>40	0 (0)
Primary site	
Oral cavity	28 (38.9)
Oropharynx	21 (29.2)
Larynx	18 (25)
Hypopharynx	4 (5.6)
Unknown primary	1 (1.4)
Stage	
1	6 (8.3)
2	9 (12.5)
3	12 (16.7)
4	45 (62.5)
Comorbidities	
Neurocognitive disorder	1 (1.4)
Substance abuse disorder	5 (6.9)
Chronic pain	10 (13.9)
Alcohol use	64 (88.9)
Tobacco use	67 (93.1)
Mental health disorder	37 (51.4)
Housing problems	3 (4.2)
Diabetes	8 (11.1)
Additional performed procedures	
Dental extraction	29 (40.3)
Gastrostomy tube placement	35 (48.6)
Prophylactic tracheostomy	4 (5.6)
Neck dissection	60 (83.3)

Abbreviation: BMI, body mass index.

**Figure 1:**
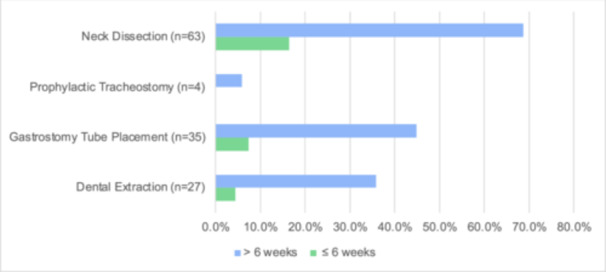
Factors influencing delays in postoperative radiation therapy.

Of the 72 veterans who received PORT, only 18 (25%) of these veterans started PORT within 6 weeks of their initial surgery. The average time from surgery to radiation was 52 (7.4 weeks) ± 21 days (interquartile range [IQR]: 42‐71 days). A multivariate analysis was conducted and 3 key features were identified to be associated with delay in PORT initiation beyond 6 weeks: neck dissection during the index surgery, undergoing preradiation dental extraction, and gastrostomy tube (G‐tube) placement (Table 2, Figure 1). Specifically, as demonstrated in Table 3, veterans who underwent neck dissection were 4 times more likely to experience delays in initiating PORT (odds ratio [OR]: 4.00, 95% confidence interval [CI]: 1.09‐14.62, *P* = .028). Those who underwent dental extraction were 3.77 times more likely to have delayed PORT (OR: 3.77, 95% CI: 1.10‐12.93, *P* = .032), and G‐tube placement was associated with a 3.25 times higher likelihood of delay (OR: 3.25, 95% CI: 1.02‐10.40, *P* = .041).

**Table 2:** Comparison of Patients Who Received PORT Within 6 Weeks Versus Beyond 6 Weeks

	No. of patients, %		
Patient variable	Initiation of PORT ≤6 wk (n = 18)	Initiation of PORT >6 wk (n = 54)	*P* value
Sex			.552
Male	18 (100)	52 (96.3)	
Female	0 (0)	2 (3.7)	
Race			.785
White	9 (50)	25 (46.3)	
Black	9 (50)	29 (53.7)	
Other			
Age, y			.797
<40	0 (0)	0 (0)	
40‐49	1 (5.6)	3 (5.6)	
50‐59	6 (33.3)	23 (42.6)	
60‐69	7 (38.9)	38.9 (33.3)	
70‐79	4 (22.2)	9 (16.7)	
>80	0 (0)	1 (1.9)	
BMI			.732
<18.5	3 (16.7)	8 (14.8)	
18.5‐24.9	9 (50)	20 (37.0)	
25‐29.9	4 (22.2)	19 (35.2)	
30‐34.9	2 (11.1)	5 (9.3)	
35‐39.9	0 (0)	2 (3.7)	
>40	0 (0)	0 (0)	
Primary site			.934
Oral cavity	6 (33.3)	22 (40.7)	
Oropharynx	6 (33.3)	15 (27.8)	
Larynx	5 (27.8)	13 (24.1)	
Hypopharynx	8 (44.4)	3 (5.6)	
Unknown primary	0 (0)	1 (1.9)	
Stage			.171
1	3 (16.7)	3 (5.6)	
2	2 (11.1)	7 (13)	
3	5 (27.8)	7 (13)	
4	8 (44.4)	37 (68.5)	
Comorbidities			
Neurocognitive disorder	0 (0.0)	1 (1.9)	.561
Mental health disorder	10 (55.6)	27 (50)	.683
Housing problems	2 (11.1)	1 (1.9)	.089
Substance abuse disorder	1 (5.6)	4 (7.4)	.789
Chronic pain	2 (11.1)	8 (14.8)	.694
Alcohol use	16 (88.9)	48 (88.9)	1.000
Tobacco use	17 (94.4)	50 (92.6)	1.000
Additional performed procedures			
Dental extraction	4 (22.2)	28 (51.9)	.032^a^
Gastrostomy tube placement	5 (27.8)	30 (55.6)	.041^a^
Prophylactic tracheostomy	0 (0.0)	4 (7.4)	.326
Neck dissection	12 (66.7)	48 (88.9)	.028^a^
Treatment type			
Surgery + adjuvant	6 (33.3)	22 (40.7)	.577
Radiation (n = 28)			
Surgery + adjuvant	12 (66.7)	32 (59.3)	.577
Chemoradiation (n = 44)			

Abbreviations: BMI, body mass index; PORT, postoperative radiation therapy.

^a^ Statistical significance.

**Table 3:** Factors Associated With Delays in Postoperative Radiation Therapy Initiation

Factors	Odds ratio	95% Confidence interval	*P* value
Neck dissection	4.00	1.09‐14.62	.028
Dental extraction	3.77	1.10‐12.93	.032
Gastrostomy tube placement	3.25	1.02‐10.40	.041

Of the patients receiving timely PORT, 66.7% underwent neck dissection, 22.2% underwent dental extractions, and 27.8% underwent G‐tube placement. Of the patients receiving delayed PORT, 88.99% underwent neck dissection (*P* = .028), 51.9% underwent dental extraction (*P* = .032), and 27.8% underwent G‐tube placement (*P* = .041). The medial interval from surgery to radiation for patients who underwent neck dissection was 52 days (IQR: 43‐68), for dental extractions was 56 days (IQR: 48‐80) and for G‐tube placements was 54 days (IQR: 46‐71).

Contrasting with prior studies, many demographic factors including age, race, sex, primary cancer site, clinical stage, and select comorbidities (dementia, mental health disorders, housing insecurity, substance use disorder, alcohol use, tobacco use, and chronic pain) were not associated with delays in PORT in this patient population.

## Discussion

Compliance with guidelines for PORT is an important indicator of quality care and results in improved survival outcomes for patients with HNSCC. In this cohort, only 25% of veterans initiated PORT within the 6‐week timeframe recommended by the NCCN guidelines. This starkly contrasts with the previously reported rate of 44% in the general population and may indicate an increased risk for delays in treatment among veterens.^6^ While both cohorts demonstrate a significant deviation from NCCN guidelines for head and neck cancer treatment, a more alarming and stark difference was observed in this veteran population.

Three key procedures were associated with failure to initiate PORT in accordance with NCCN guidelines. In this study, veterans who underwent neck dissection, dental extractions, or G‐tube placements were more likely to start PORT in a delayed fashion. Patients who require neck dissections are more likely to experience PORT delay, possibly due to increased risk of postoperative complications leading to prolonged hospitalizations or unplanned readmissions.^9^ Similarly, patients who require G‐tube placement may also require longer inpatient stays due to the additional time required to coordinate swallow evaluations and interventional radiology or gastroenterology consultation. The added challenge of arranging home nursing, supplies, and tube feeds for the patient after discharge may also delay discharge and therefore PORT. Prior studies have indeed shown that patients who receive G‐tubes experience longer intensive care unit stays and thus prolonged hospitalizations.^10^ Although our study did not explicitly evaluate length of hospitalization, neck dissection and G‐tube placement may serve as a surrogate for this variable.

In our study, patients who underwent dental extractions prior to radiation were more likely to experience delays in starting PORT (*P* = .032). Dental providers often consider extracting teeth in the proposed radiation field that are affected by periodontal disease, dental caries, root canals, impactions, large fillings or fractures, or those that are unopposed.^11^ Most dental providers and radiation oncologists recommend waiting a minimum of 14 to 21 days after extractions before proceeding with radiation to prevent the feared complication of osteoradionecrosis.^11^ Given the time necessary to heal after extractions, delays in coordinating dental care may subject patients to delays in initiating radiation. This concern is not unique to veterans; a study from Stanford evaluating patients with oral cavity HNSCC who underwent surgery followed by PORT similarly found that delayed dental extractions were one of several key drivers for delayed PORT.^12^ As part of a multistep quality improvement initiative, this group implemented Panorex scans and formal dental consultations preoperatively, allowing for all necessary extractions to be performed prior to or during surgical resection of their patients' cancer. These interventions combined with other measures resulted in a significant improvement in delivery of timely PORT. Similarly, Strohl et al found that patients who received dental extractions prior to or during the index surgery were less likely to experience delays in PORT compared to patients who received extractions after surgery.^13^

Fragmentation of care including the use of different facilities for surgery and radiation, difficulty obtaining inpatient radiation oncology or dental consults, or difficulty in scheduling postoperative referrals, may also serve as potential barriers to the delivery of timely adjuvant treatment. While our study did not specifically address whether these issues had an impact on timing of PORT, it is important to consider how care fragmentation might affect the veteran population and work to address systemic issues that may contribute to this. In the authors' experience, for example, many patients who receive care at our facility travel from several hours away to undergo surgery, which may be prohibitive to daily radiation. These patients may elect to undergo radiation closer to their homes at non‐VA facilities, which may impact their ability to receive timely care.

The small sample size in this retrospective cohort analysis has been considered an important limitation of this single‐institution study and likely reflects both a clerical underreporting of the data in the tumor registry and an extensive loss to follow‐up.^14^ These factors were difficult to precisely investigate given that the researchers collecting data from the tumor registry did not have complete information regarding the individual patient understanding of the risks/benefits to care and ability to meet his/her appointment schedule ‐ likely affecting compliance to follow‐up for PORT. Additionally, factors not included in the registry, such as duration of hospitalization, postoperative complications, and use of free tissue transfer or other reconstruction methods, can influence outcomes and act as potential confounding variables. The delivery of radiotherapy at VA facilities versus other locations, as well as the unrecorded distance from home, can further complicate data analysis. While this finding hampers the generalizability of a portion of this data, it importantly identifies the need for future studies to understand the institutional shortcomings of systematic reporting of veterans with oncologic pathologies.

Despite these limitations, the study was able to determine specific factors ‐ neck dissection, dental extraction, and G‐tube placement—impeding NCCN guideline‐adherent PORT within 6 weeks of surgery in veterans. This information provides an opportunity to address these factors to better comply with NCCN guideline‐adherent PORT and ultimately promote improved survival and decreased risk of recurrence in these populations.

## Conclusion

This study determined that a minority of veterans at the DC VAMC received NCCN guideline‐adherent PORT within 6 weeks of surgery. Specific procedures including neck dissection, dental extraction, and G‐tube placement, were found to be associated with delays in initiation of PORT in the veteran population. Prior studies have shown that receiving PORT within 6 weeks of surgery is associated with improved survival and decreased risk of recurrence.^3^, ^6^ It is incumbent upon health care practitioners to note the suboptimal adherence rates within this patient cohort and exert every effort to ensure the timely administration of adjuvant treatment, as sustained endeavors are essential to enhance the quality of care provided to our veteran patient population.

## Author Contributions

All authors have contributed equally to this manuscript.

## Disclosures

### Competing interests

The authors have no relevant conflicts of interest to report.

### Funding source

There are no funding sources to report.

## References

[1] American Cancer Society . Cancer Facts & Figures 2022. 2022. Accessed October 7, 2023. https://www.cancer.org/content/dam/cancer-org/research/cancer-facts-and-statistics/annual-cancer-facts-and-figures/2020/cancer-facts-and-figures-2020.pdf

[2] National Comprehensive Cancer Network . NCCN Guidelines Version 2.2020: Head and Neck Cancers. 1995. Accessed August 5, 2023. https://www.nccn.org/professionals/physician_gls/pdf/head-and-neck.pdf

[3] Graboyes EM , Kompelli AR , Neskey DM , et al. Association of treatment delays with survival for patients with head and neck cancer: a systematic review. JAMA Otolaryngol Head Neck Surg. 2019;145(2):166‐177. 10.1001/jamaoto.2018.2716 30383146 PMC6494704

[4] Al‐Dweri FMO , Guirado D , Lallena AM , Pedraza V . Effect on tumour control of time interval between surgery and postoperative radiotherapy: an empirical approach using Monte Carlo simulation. Phys Med Biol. 2004;49(13):2827‐2839. 10.1088/0031-9155/49/13/005 15285250

[5] Mackillop WJ , Bates JHT , O'Sullivan B , Withers HR . The effect of delay in treatment on local control by radiotherapy. Int J Radiat Oncol Biol Phys. 1996;34(1):243‐250. 10.1016/0360-3016(95)02049-7 12118558

[6] Graboyes EM , Garrett‐Mayer E , Sharma AK , Lentsch EJ , Day TA . Adherence to National Comprehensive Cancer Network guidelines for time to initiation of postoperative radiation therapy for patients with head and neck cancer. Cancer. 2017;123(14):2651‐2660. 10.1002/cncr.30651 28241092

[7] Liao DZ , Schlecht NF , Rosenblatt G , et al. Association of delayed time to treatment initiation with overall survival and recurrence among patients with head and neck squamous cell carcinoma in an underserved urban population. JAMA Otolaryngol Head Neck Surg. 2019;145(11):1001‐1009. 10.1001/jamaoto.2019.2414 31513264 PMC6743055

[8] Bilimoria KY , Ko CY , Tomlinson JS , et al. Wait times for cancer surgery in the United States. Ann Surg. 2011;253(4):779‐785. 10.1097/sla.0b013e318211cc0f 21475020

[9] Lorenz FJ , Mahase SS , Miccio J , King TS , Pradhan S , Goyal N . Update on adherence to guidelines for time to initiation of postoperative radiation for head and neck squamous cell carcinoma. Head Neck. 2023;45(7):1676‐1691. 10.1002/hed.27380 37102787 PMC10797635

[10] Mays AC , Worley M , Ackall F , D'Agostino Jr, R , Waltonen JD . The association between gastrostomy tube placement, poor post‐operative outcomes, and hospital re‐admissions in head and neck cancer patients. Surg Oncol. 2015;24(3):248‐257. 10.1016/j.suronc.2015.08.005 26321115 PMC4669044

[11] Devi S , Singh N . Dental care during and after radiotherapy in head and neck cancer. Natl J Maxillofac Surg. 2014;5(2):117‐125. 10.4103/0975-5950.154812 25937720 PMC4405951

[12] Divi V , Chen MM , Hara W , et al. Reducing the time from surgery to adjuvant radiation therapy: an institutional quality improvement project. Otolaryngol Head Neck Surg. 2018;159(1):158‐165. 10.1177/0194599818768254 29631478

[13] Strohl MP , Chen JP , Ha PK , Seth R , Yom SS , Heaton CM . Can early dental extractions reduce delays in postoperative radiation for patients with advanced oral cavity carcinoma? J Oral Maxillofac Surg. 2019;77(11):2215‐2220. 10.1016/j.joms.2019.05.007 31228426

[14] Graboyes EM , Garrett‐Mayer E , Ellis MA , et al. Effect of time to initiation of postoperative radiation therapy on survival in surgically managed head and neck cancer. Cancer. 2017;123(24):4841‐4850. 10.1002/cncr.30939 28841234 PMC5759768

